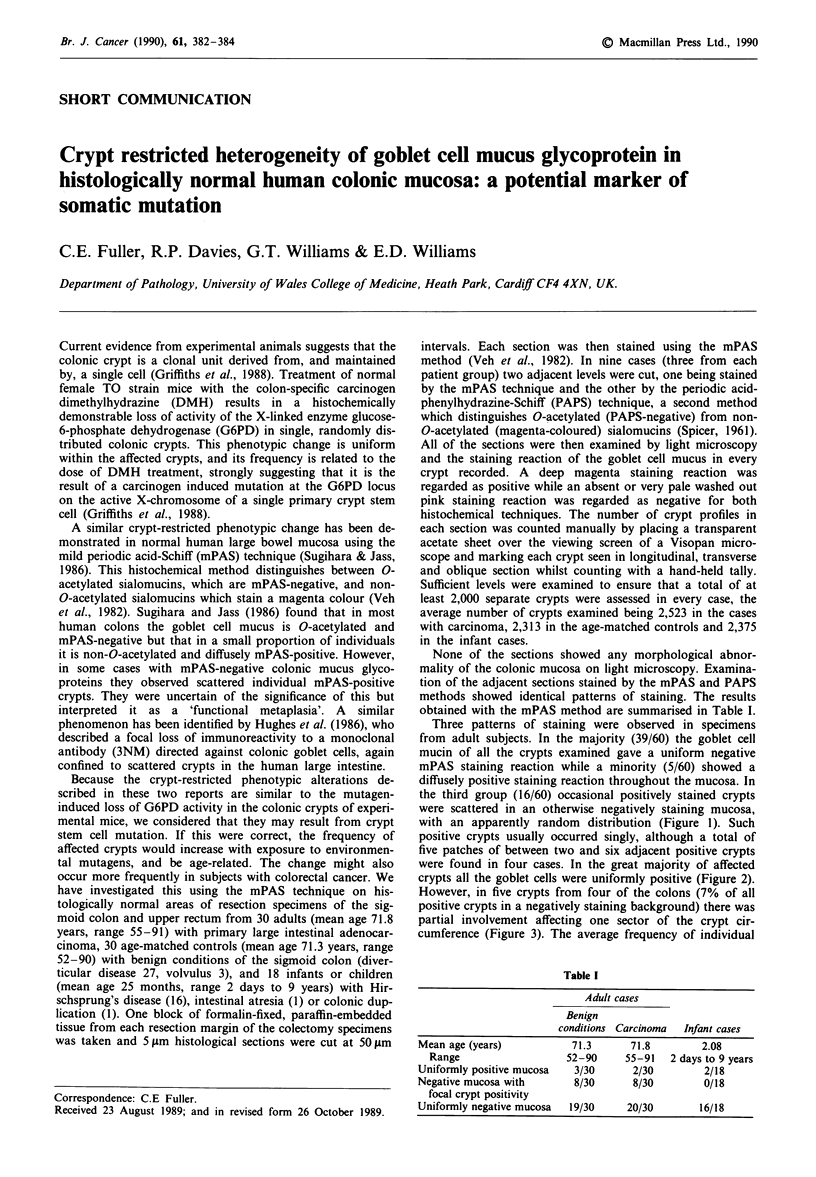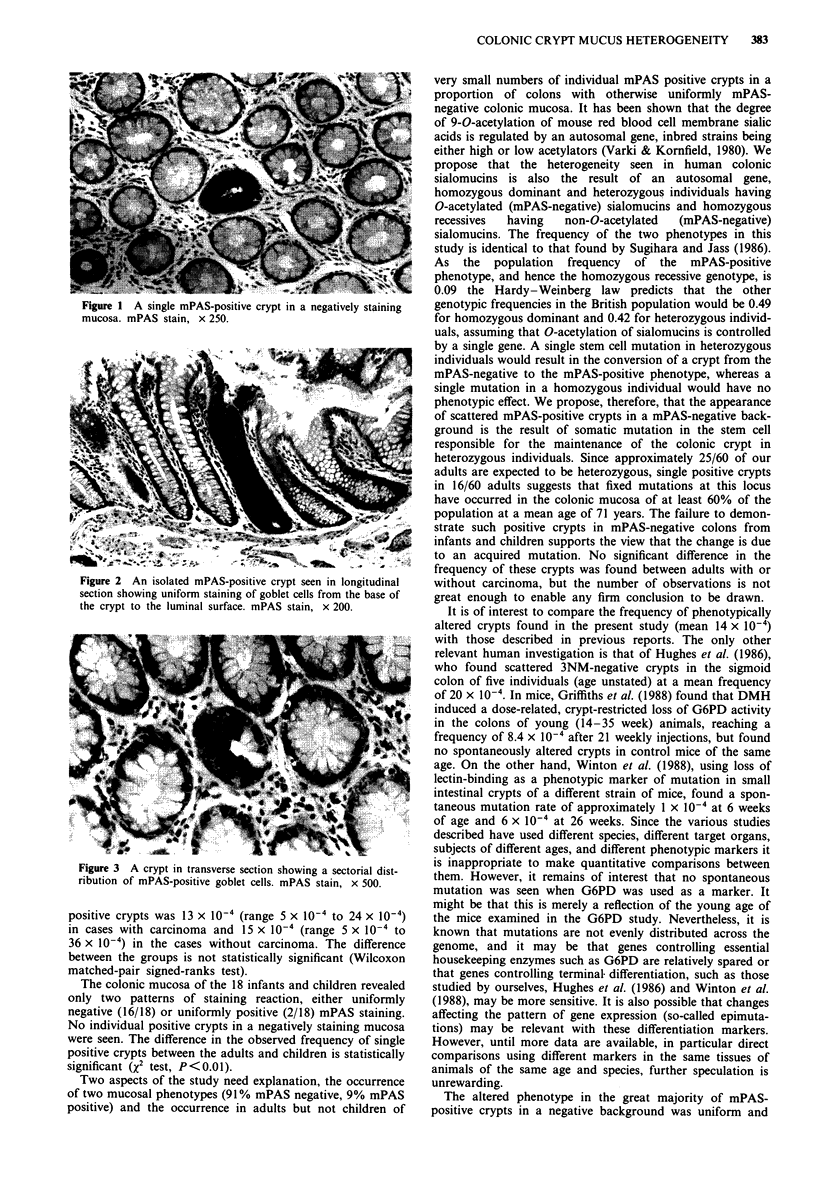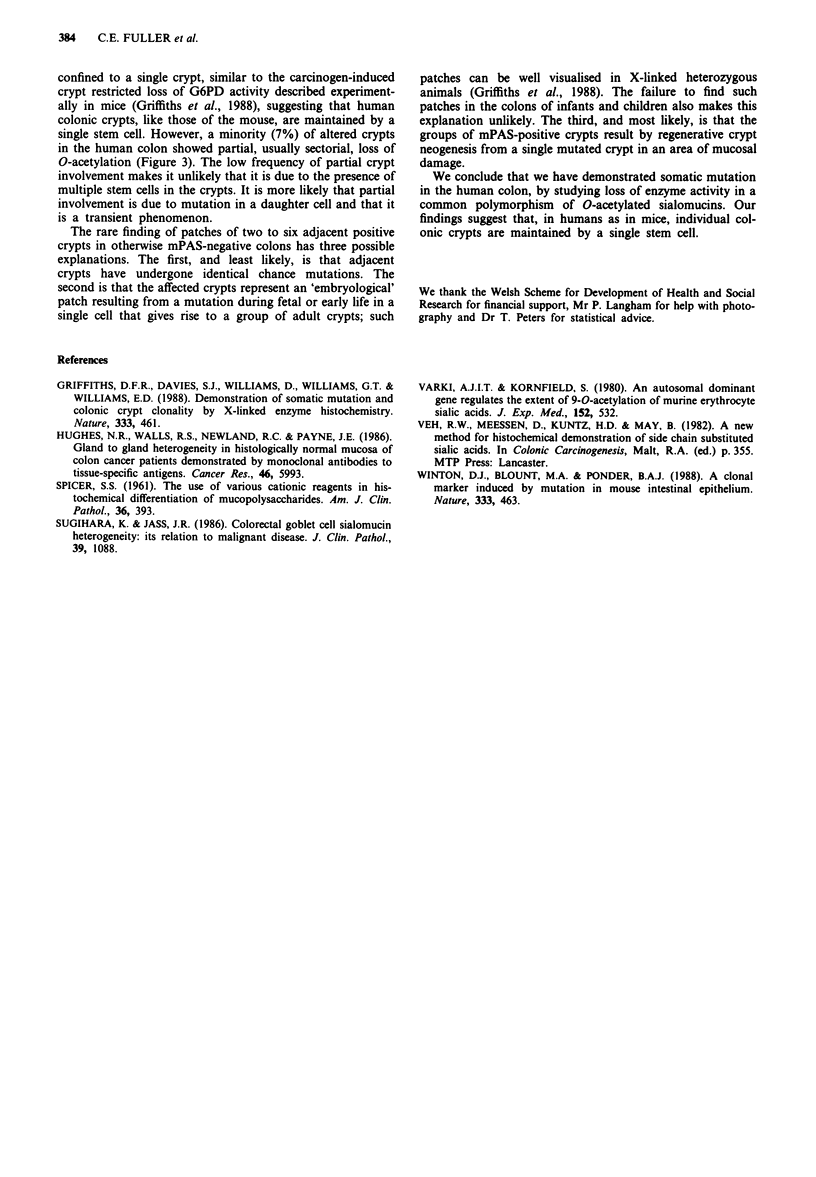# Crypt restricted heterogeneity of goblet cell mucus glycoprotein in histologically normal human colonic mucosa: a potential marker of somatic mutation.

**DOI:** 10.1038/bjc.1990.83

**Published:** 1990-03

**Authors:** C. E. Fuller, R. P. Davies, G. T. Williams, E. D. Williams

**Affiliations:** Department of Pathology, University of Wales College of Medicine, Heath Park, Cardiff, UK.

## Abstract

**Images:**


Br. J. Cancer (1990), 61, 382-384 © Macmillan Press Ltd., 1990

SHORT COMMUNICATION

### Crypt restricted heterogeneity of goblet cell mucus glycoprotein in histologically normal human colonic mucosa: a potential marker of somatic mutation

C.E. Fuller, R.P. Davies, G.T. Williams & E.D. Williams

*Department of Pathology, University of Wales College of Medicine, Heath Park, Cardiff CF4 4XN, UK.*

Current evidence from experimental animals suggests that the colonic crypt is a clonal unit derived from, and maintained by, a single cell (Griffiths *et al.*, 1988). Treatment of normal female TO strain mice with the colon-specific carcinogen dimethylhydrazine (DMH) results in a histochemically demonstrable loss of activity of the X-linked enzyme glucose-6-phosphate dehydrogenase (G6PD) in single, randomly distributed colonic crypts. This phenotypic change is uniform within the affected crypts, and its frequency is related to the dose of DMH treatment, strongly suggesting that it is the result of a carcinogen induced mutation at the G6PD locus on the active X-chromosome of a single primary crypt stem cell (Griffiths *et al.*, 1988).

A similar crypt-restricted phenotypic change has been demonstrated in normal human large bowel mucosa using the mild periodic acid-Schiff (mPAS) technique (Sugihara & Jass, 1986). This histochemical method distinguishes between *O*-acetylated sialomucins, which are mPAS-negative, and non-*O*-acetylated sialomucins which stain a magenta colour (Veh *et al.*, 1982). Sugihara and Jass (1986) found that in most human colons the goblet cell mucus is *O*-acetylated and mPAS-negative but that in a small proportion of individuals it is non-*O*-acetylated and diffusely mPAS-positive. However, in some cases with mPAS-negative colonic mucus glycoproteins they observed scattered individual mPAS-positive crypts. They were uncertain of the significance of this but interpreted it as a 'functional metaplasia'. A similar phenomenon has been identified by Hughes *et al.* (1986), who described a focal loss of immunoreactivity to a monoclonal antibody (3NM) directed against colonic goblet cells, again confined to scattered crypts in the human large intestine.

Because the crypt-restricted phenotypic alterations described in these two reports are similar to the mutagen-induced loss of G6PD activity in the colonic crypts of experimental mice, we considered that they may result from crypt stem cell mutation. If this were correct, the frequency of affected crypts would increase with exposure to environmental mutagens, and be age-related. The change might also occur more frequently in subjects with colorectal cancer. We have investigated this using the mPAS technique on histologically normal areas of resection specimens of the sigmoid colon and upper rectum from 30 adults (mean age 71.8 years, range 55-91) with primary large intestinal adenocarcinoma, 30 age-matched controls (mean age 71.3 years, range 52-90) with benign conditions of the sigmoid colon (diverticular disease 27, volvulus 3), and 18 infants or children (mean age 25 months, range 2 days to 9 years) with Hirschsprung's disease (16), intestinal atresia (1) or colonic duplication (1). One block of formalin-fixed, paraffin-embedded tissue from each resection margin of the colectomy specimens was taken and 5 μm histological sections were cut at 50 μm intervals. Each section was then stained using the mPAS method (Veh *et al.*, 1982). In nine cases (three from each patient group) two adjacent levels were cut, one being stained by the mPAS technique and the other by the periodic acid-phenylhydrazine-Schiff (PAPS) technique, a second method which distinguishes *O*-acetylated (PAPS-negative) from non-*O*-acetylated (magenta-coloured) sialomucins (Spicer, 1961). All of the sections were then examined by light microscopy and the staining reaction of the goblet cell mucus in every crypt recorded. A deep magenta staining reaction was regarded as positive while an absent or very pale washed out pink staining reaction was regarded as negative for both histochemical techniques. The number of crypt profiles in each section was counted manually by placing a transparent acetate sheet over the viewing screen of a Visopan microscope and marking each crypt seen in longitudinal, transverse and oblique section whilst counting with a hand-held tally. Sufficient levels were examined to ensure that a total of at least 2,000 separate crypts were assessed in every case, the average number of crypts examined being 2,523 in the cases with carcinoma, 2,313 in the age-matched controls and 2,375 in the infant cases.

None of the sections showed any morphological abnormality of the colonic mucosa on light microscopy. Examination of the adjacent sections stained by the mPAS and PAPS methods showed identical patterns of staining. The results obtained with the mPAS method are summarised in Table I.

Three patterns of staining were observed in specimens from adult subjects. In the majority (39/60) the goblet cell mucin of all the crypts examined gave a uniform negative mPAS staining reaction while a minority (5/60) showed a diffusely positive staining reaction throughout the mucosa. In the third group (16/60) occasional positively stained crypts were scattered in an otherwise negatively staining mucosa, with an apparently random distribution (Figure 1). Such positive crypts usually occurred singly, although a total of five patches of between two and six adjacent positive crypts were found in four cases. In the great majority of affected crypts all the goblet cells were uniformly positive (Figure 2). However, in five crypts from four of the colons (7% of all positive crypts in a negatively staining background) there was partial involvement affecting one sector of the crypt circumference (Figure 3). The average frequency of individual

**Table I**

| | *Adult cases* | | |
|---|---|---|---|
| | *Benign conditions* | *Carcinoma* | *Infant cases* |
| Mean age (years) | 71.3 | 71.8 | 2.08 |
| Range | 52-90 | 55-91 | 2 days to 9 years |
| Uniformly positive mucosa | 3/30 | 2/30 | 2/18 |
| Negative mucosa with focal crypt positivity | 8/30 | 8/30 | 0/18 |
| Uniformly negative mucosa | 19/30 | 20/30 | 16/18 |

Correspondence: C.E Fuller.
Received 23 August 1989; and in revised form 26 October 1989.

**Figure 1** A single mPAS-positive crypt in a negatively staining mucosa. mPAS stain, × 250.

**Figure 2** An isolated mPAS-positive crypt seen in longitudinal section showing uniform staining of goblet cells from the base of the crypt to the luminal surface. mPAS stain, × 200.

**Figure 3** A crypt in transverse section showing a sectorial distribution of mPAS-positive goblet cells. mPAS stain, × 500.

positive crypts was $13 \times 10^{-4}$ (range $5 \times 10^{-4}$ to $24 \times 10^{-4}$) in cases with carcinoma and $15 \times 10^{-4}$ (range $5 \times 10^{-4}$ to $36 \times 10^{-4}$) in the cases without carcinoma. The difference between the groups is not statistically significant (Wilcoxon matched-pair signed-ranks test).

The colonic mucosa of the 18 infants and children revealed only two patterns of staining reaction, either uniformly negative (16/18) or uniformly positive (2/18) mPAS staining. No individual positive crypts in a negatively staining mucosa were seen. The difference in the observed frequency of single positive crypts between the adults and children is statistically significant ($\chi^2$ test, $P < 0.01$).

Two aspects of the study need explanation, the occurrence of two mucosal phenotypes (91% mPAS negative, 9% mPAS positive) and the occurrence in adults but not children of very small numbers of individual mPAS positive crypts in a proportion of colons with otherwise uniformly mPAS-negative colonic mucosa. It has been shown that the degree of 9-*O*-acetylation of mouse red blood cell membrane sialic acids is regulated by an autosomal gene, inbred strains being either high or low acetylators (Varki & Kornfield, 1980). We propose that the heterogeneity seen in human colonic sialomucins is also the result of an autosomal gene, homozygous dominant and heterozygous individuals having *O*-acetylated (mPAS-negative) sialomucins and homozygous recessives having non-*O*-acetylated (mPAS-negative) sialomucins. The frequency of the two phenotypes in this study is identical to that found by Sugihara and Jass (1986). As the population frequency of the mPAS-positive phenotype, and hence the homozygous recessive genotype, is 0.09 the Hardy–Weinberg law predicts that the other genotypic frequencies in the British population would be 0.49 for homozygous dominant and 0.42 for heterozygous individuals, assuming that *O*-acetylation of sialomucins is controlled by a single gene. A single stem cell mutation in heterozygous individuals would result in the conversion of a crypt from the mPAS-negative to the mPAS-positive phenotype, whereas a single mutation in a homozygous individual would have no phenotypic effect. We propose, therefore, that the appearance of scattered mPAS-positive crypts in a mPAS-negative background is the result of somatic mutation in the stem cell responsible for the maintenance of the colonic crypt in heterozygous individuals. Since approximately 25/60 of our adults are expected to be heterozygous, single positive crypts in 16/60 adults suggests that fixed mutations at this locus have occurred in the colonic mucosa of at least 60% of the population at a mean age of 71 years. The failure to demonstrate such positive crypts in mPAS-negative colons from infants and children supports the view that the change is due to an acquired mutation. No significant difference in the frequency of these crypts was found between adults with or without carcinoma, but the number of observations is not great enough to enable any firm conclusion to be drawn.

It is of interest to compare the frequency of phenotypically altered crypts found in the present study (mean $14 \times 10^{-4}$) with those described in previous reports. The only other relevant human investigation is that of Hughes *et al.* (1986), who found scattered 3NM-negative crypts in the sigmoid colon of five individuals (age unstated) at a mean frequency of $20 \times 10^{-4}$. In mice, Griffiths *et al.* (1988) found that DMH induced a dose-related, crypt-restricted loss of G6PD activity in the colons of young (14–35 week) animals, reaching a frequency of $8.4 \times 10^{-4}$ after 21 weekly injections, but found no spontaneously altered crypts in control mice of the same age. On the other hand, Winton *et al.* (1988), using loss of lectin-binding as a phenotypic marker of mutation in small intestinal crypts of a different strain of mice, found a spontaneous mutation rate of approximately $1 \times 10^{-4}$ at 6 weeks of age and $6 \times 10^{-4}$ at 26 weeks. Since the various studies described have used different species, different target organs, subjects of different ages, and different phenotypic markers it is inappropriate to make quantitative comparisons between them. However, it remains of interest that no spontaneous mutation was seen when G6PD was used as a marker. It might be that this is merely a reflection of the young age of the mice examined in the G6PD study. Nevertheless, it is known that mutations are not evenly distributed across the genome, and it may be that genes controlling essential housekeeping enzymes such as G6PD are relatively spared or that genes controlling terminal differentiation, such as those studied by ourselves, Hughes *et al.* (1986) and Winton *et al.* (1988), may be more sensitive. It is also possible that changes affecting the pattern of gene expression (so-called epimutations) may be relevant with these differentiation markers. However, until more data are available, in particular direct comparisons using different markers in the same tissues of animals of the same age and species, further speculation is unrewarding.

The altered phenotype in the great majority of mPAS-positive crypts in a negative background was uniform and confined to a single crypt, similar to the carcinogen-induced crypt restricted loss of G6PD activity described experimentally in mice (Griffiths *et al.*, 1988), suggesting that human colonic crypts, like those of the mouse, are maintained by a single stem cell. However, a minority (7%) of altered crypts in the human colon showed partial, usually sectorial, loss of *O*-acetylation (Figure 3). The low frequency of partial crypt involvement makes it unlikely that it is due to the presence of multiple stem cells in the crypts. It is more likely that partial involvement is due to mutation in a daughter cell and that it is a transient phenomenon.

The rare finding of patches of two to six adjacent positive crypts in otherwise mPAS-negative colons has three possible explanations. The first, and least likely, is that adjacent crypts have undergone identical chance mutations. The second is that the affected crypts represent an 'embryological' patch resulting from a mutation during fetal or early life in a single cell that gives rise to a group of adult crypts; such patches can be well visualised in X-linked heterozygous animals (Griffiths *et al.*, 1988). The failure to find such patches in the colons of infants and children also makes this explanation unlikely. The third, and most likely, is that the groups of mPAS-positive crypts result by regenerative crypt neogenesis from a single mutated crypt in an area of mucosal damage.

We conclude that we have demonstrated somatic mutation in the human colon, by studying loss of enzyme activity in a common polymorphism of *O*-acetylated sialomucins. Our findings suggest that, in humans as in mice, individual colonic crypts are maintained by a single stem cell.

We thank the Welsh Scheme for Development of Health and Social Research for financial support, Mr P. Langham for help with photography and Dr T. Peters for statistical advice.

### References

GRIFFITHS, D.F.R., DAVIES, S.J., WILLIAMS, D., WILLIAMS, G.T. & WILLIAMS, E.D. (1988). Demonstration of somatic mutation and colonic crypt clonality by X-linked enzyme histochemistry. *Nature*, **333**, 461.

HUGHES, N.R., WALLS, R.S., NEWLAND, R.C. & PAYNE, J.E. (1986). Gland to gland heterogeneity in histologically normal mucosa of colon cancer patients demonstrated by monoclonal antibodies to tissue-specific antigens. *Cancer Res.*, **46**, 5993.

SPICER, S.S. (1961). The use of various cationic reagents in histochemical differentiation of mucopolysaccharides. *Am. J. Clin. Pathol.*, **36**, 393.

SUGIHARA, K. & JASS, J.R. (1986). Colorectal goblet cell sialomucin heterogeneity: its relation to malignant disease. *J. Clin. Pathol.*, **39**, 1088.

VARKI, A.J.I.T. & KORNFIELD, S. (1980). An autosomal dominant gene regulates the extent of 9-*O*-acetylation of murine erythrocyte sialic acids. *J. Exp. Med.*, **152**, 532.

VEH, R.W., MEESSEN, D., KUNTZ, H.D. & MAY, B. (1982). A new method for histochemical demonstration of side chain substituted sialic acids. In *Colonic Carcinogenesis*, Malt, R.A. (ed.) p. 355. MTP Press: Lancaster.

WINTON, D.J., BLOUNT, M.A. & PONDER, B.A.J. (1988). A clonal marker induced by mutation in mouse intestinal epithelium. *Nature*, **333**, 463.